# Validation of EORTC, CUETO, and EAU risk stratification in prediction of recurrence, progression, and death of patients with initially non–muscle‐invasive bladder cancer (NMIBC): A cohort analysis

**DOI:** 10.1002/cam4.3007

**Published:** 2020-03-26

**Authors:** Mateusz Jobczyk, Konrad Stawiski, Wojciech Fendler, Waldemar Różański

**Affiliations:** ^1^ Department of Urology Copernicus Memorial Hospital Medical University of Lodz Lodz Poland; ^2^ Department of Urology The Hospital Ministry of the Interior and Administration Lodz Poland; ^3^ Department of Biostatistics and Translational Medicine Medical University of Lodz Lodz Poland; ^4^ Department of Radiation Oncology Dana‐Farber Cancer Institute Harvard Medical School Boston MA USA

**Keywords:** bladder cancer, non–muscle‐invasive bladder cancer, prediction, risk stratification, systematic review

## Abstract

Brief Description: The results demonstrate that the European Organisation for Research and Treatment of Cancer (EORTC) scale provides the best recurrence and progression prediction in comparison with European Association of Urology (EAU) and Club Urologico Espanol de Tratamiento Oncologico (CUETO) risk scores among a mixed population of patients with non–muscle‐invasive bladder who were treated with, or without, Bacillus Calmette‐Guerin (BCG) and without any immediate postoperative chemotherapy. The study highlights the role of tumor diameter and extent in transition prediction.

This retrospective cohort analysis of 322 patients with newly diagnosed non–muscle‐invasive bladder cancer (NMIBC) assesses the concordance and accuracy of predicting recurrence and progression by EAU‐recommended tools (EAU risk groups, EORTC, and CUETO). One‐year and five‐year c‐indices ranged from 0.55 to 0.66 for recurrence and from 0.72 to 0.82 for progression. AUCROC of predictions ranged from 0.46 for 1‐year recurrence risk based on CUETO groups, to 0.82 for 1‐year progression risk based on EAU risk groups. Diameter (HR: 1.91; 95% CI: 1.39‐2.61) and tumor extent (HR: 1.21; 95% CI: 1.01‐1.46 for recurrence; HR: 3.1; 95% CI: 1.40‐6.87 for progression) were shown to be significant predictors in multistate analysis. Lower accuracy of prediction was observed for patients treated with BCG maintenance immunotherapy. The EORTC model (overall c‐index c = 0.64; 95% CI: 0.61‐0.68) was superior to the EAU (*P* = .035; .62; 95% CI: 0.59‐0.66) and CUETO (*P* < .001; c = 0.53; 95% CI: 0.50‐0.56) models in predicting recurrence. The EORTC model (c = 0.82; 95% CI: 0.77‐0.86) also performed better than CUETO (*P* = .008; c = 0.73; 95% CI: 0.66‐0.81) but there was no sufficient evidence that it performed better than EAU (*P* = .572; c = 0.81; 95% CI: 0.77‐0.84) for predicting progression. EORTC and CUETO gave similar predictions for progression in BCG‐treated EAU high‐risk patients (*P* = .48). We share anonymized individual patient data. In conclusion, despite moderate accuracy, EORTC provided the best recurrence and progression prediction for a mixed population of patients treated with, or without BCG, and without immediate postoperative chemotherapy.

## Graphical abstract

For mixed population of patients with non–muscle‐invasive bladder who were treated with and without Bacillus Calmette‐Guerin and without any immediate postoperative chemotherapy, we show that European Organisation for Research and Treatment of Cancer scale provides the best recurrence and progression prediction in comparison with European Association of Urology and Club Urologico Espanol de Tratamiento Oncologico risk scores. We highlight the role of diameter and tumor extent in transition prediction.

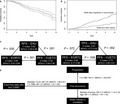

## 1 INTRODUCTION

According to GLOBOCAN, bladder cancer (BC) is the most common malignancy of the urinary tract. It remains the seventh most common cancer in men and the seventeenth in women. In the European Union, the age‐standardized incidence rate is 27 per 100 000 in men and 6 per 100 000 in women.

In general, 75% of newly diagnosed bladder cancer cases are non–muscle‐invasive (NMIBC) forms, which are characterized by a high rate of recurrence and progression, despite local treatment.^1^ This requires patients to follow a regular schedule of visits and conduction of many potentially superfluous procedures (as cystoscopy). The remaining 25% are of the muscle‐invasive type (MIBC). As MIBC needs radical treatment (cystectomy, radiotherapy, and chemotherapy), prediction of recurrence and progression from NMIBC to MIBC remains a perennial topic of research.^2^

NMIBC is generally associated with 5‐year survival higher than 88%,^3^ however, up to 70% of NMIBC tumors recur after initial treatment, and are associated with 10%‐20% lifetime risk of progression to MIBC.^4^ In case of MIBC, the prognosis is much more unfavorable, as the 5‐year survival rate ranges from 63% to as low as 15%.^3^ Thus, in 2006 EORTC (European Organisation for Research and Treatment of Cancer) developed a risk stratification tool to predict 1‐ and 5‐year probability of recurrence and progression after transurethral resection of bladder tumor (TURBT).^5^ The topic was followed in 2009 by CUETO (Club Urologico Espanol de Tratamiento Oncologico), which published a comparable risk model adapted for patients receiving BCG (Bacillus Calmette‐Guerin) maintenance immunotherapy.^6^ Both the EORTC and CUETO model stratify patients into four risk groups based on a retrospective analysis of clinical trial data; these are based mainly on gender, age, tumor size and extent (defined as T in TNM staging), concomitant Tis (carcinoma in situ), grade, number of tumors, and recurrence status. Additionally, the most recent guidelines of the European Association of Urology (EAU) also define a three‐group risk stratification algorithm utilizing the same features.^2^ EAU categories reclassified about 38% patients into a higher‐risk group of recurrence and 12% into a higher risk of progression.^7^

The study on which the EORTC classification was based did not include patients treated with BCG, the CUETO only included patients with a short maintenance schedule of BCG therapy, and the EAU risk stratification is based mainly on the risk of progression, not recurrence. The universal assessment of the risk of recurrence and progression in NMIBC is, therefore, still an unsolved issue and the performance of those systems for real‐life mixed and heterogeneous cohorts remains uncertain.

Despite extensive research, those scales remain the golden standard of NMIBC risk stratification, all three are discussed in most recent EAU guidelines and none of them were proved superior to each other. The aim of this work was to validate and summarize current evidence about the reliability of EORTC, CUETO, and EAU risk stratification in the prediction of recurrence, progression, and death of patients with initially non–muscle‐invasive bladder cancer.

## 2 METHODS

This retrospective cohort analysis included patients with newly diagnosed NMIBC who were treated with transurethral resection of the bladder tumor (TURBT). All patients were admitted to the Department of Urology of The Hospital Ministry of the Interior and Administration in Lodz over a 10‐year period from January 2005 to December 2015, and were later followed until August 2017 in terms of disease recurrence, progression, or death.

The following inclusion criteria were applied during the revision process: (a) the patients had to be primarily diagnosed with urothelial bladder tumor, (b) ECOG Scale of Performance Status (PS) equaled 0 or 1 at the time of first resection (control for comorbidities), (c) first resection was performed during the accrual period from 2005 to 2015, and (d) NMIBC (Ta, Tis, or T1 stage of tumor extent) was confirmed by histopathological report following the first procedure. If the first resection was not complete, a second procedure was conducted as described below. If the muscle‐invasive type of bladder cancer (MIBC) was diagnosed (during first or second TURBT procedure), the patient was excluded from further analysis. In addition, patients were also disqualified from the analysis if initial performance status initial imaging studies showed advanced or disseminated disease (invasion of the perivesical or adjacent tissue, local or distant metastases). Exclusion criteria were met by the patients with insufficient follow‐up, that is, those who did not show up to the first follow‐up visit 3 months after the TURBT procedure. Acquired initial clinical (age at diagnosis, gender, smoking status, hematuria at diagnosis, number of tumors, and a diameter of tumor) and pathological factors (T stage according to current TNM classification, grading) were later used for risk estimation using EORTC,^5^ CUETO,^6^ and EAU risk stratification^2^ algorithms. Definitions for disease recurrence and progression followed those defined in original articles^5^, ^6^ and were also consistent with recent recommendations^8^ but the progression was defined as the presence muscle‐invasive disease (≥T2; to ascertain the consistency with previous publications).

### 2.1 TURBT procedures and follow‐up

All TURBT procedures were performed by the same team of five urologists according to standard procedure protocol and current EAU guidelines. Each procedure was supervised by the specialist with at least 5 years experience. Whole visible tumors were resected while maintaining the best possible proper margin of normal tissue. After the surgery, patients were not subjected to any immediate postoperative chemotherapy. All collected specimens were examined by a pathologist (specialist) according to the 1973 World Health Organization (WHO) classification system and staged using TNM system. The second TURBT was performed if the first resection was not complete, if T1 stage was reported, or if the presence of muscle fibers was not confirmed by a pathologist in the specimen from the high‐risk patient. Delay associated with the second resection after the TURBT had to be no longer than 6 weeks. Additional treatment with BCG maintenance could be ordered following the agreement of the doctors during a case conference.

In follow‐up, patients underwent cystoscopy every 3 months for 2 years, and then every 6 months during the following years. The procedures were performed by the same team of urologists. Next, TURBT procedure was performed in case of suspected recurrence or progression. All endpoints had to be confirmed by pathologists' reports. Overall survival data of the selected cohort were acquired upon the author's request for the Polish Ministry of Digital Affairs.

### 2.2 Statistical analysis

Intragroup associations were assessed using Pearson's Chi‐squared test (with Yates' continuity correction if appropriate), Spearman's rank correlation rho, one‐tailed one‐sample proportions test, unpaired and paired *t* test, and Wilcoxon rank sum test with continuity correction. Survival analysis was conducted using Kaplan‐Meier estimate with analysis using univariate Cox's proportional hazards model, as well as the log‐rank test. In the modeling, the correlation between Schoenfeld residuals and (transformed) time using a Chi‐square test was assessed as part of assumptions testing. The concordance with EORTC, CUETO, and EAU risk stratification groups was estimated using Harrell's c‐index for right‐censored event times, with a value of 1.0 indicating the perfect concordance. Weighted one‐sample *t* test has been used to compare the differences in means of Sommer's d—comparing the concordance of Cox's proportional hazards models utilizing different risk stratification models. Mean Sommer's d value and its confidence interval were converted into Harrell's c‐index. The predictive ability of those algorithms was additionally assessed using the area under receiver operating characteristic curve (AUC ROC) for prespecified periods of 1 and 5 years since first TURBT procedure. Estimated cumulative incidences were calculated for multistate outcomes, including death as a competing risk. Multistate Cox‐Markov model was performed to describe the influence of specific risk factors on transitions between event‐free, after first recurrence, after progression states, and death, as well as to elude the difference between the risk of death after recurrence or progression and the death from other or unknown cause. Thus, estimated cumulative incidences were calculated for multistate outcomes.^9^ None of the missing data was crucial to the analysis, thus no data were imputed. All statistical analyses were performed using STATISTICA 13 (TIBCO software) and R statistical programming language.

### 2.3 Systematic review

Our results were finally presented in the context of previous research by conducting the systematic review based on Ovid MEDLINE database. The query search was constructed as follows: (*"EORTC" OR "CUETO" OR "EAU") AND non‐muscle‐invasive bladder cancer AND ("progression" or "recurrence" or "survival"*). This part of the analysis followed PRISMA guidelines and included the screening and full‐text analysis by two authors (MJ and KS). The reasons for exclusion during screening were another study question, another study group, and a review. The date of last search was 2018‐10‐31. The studies were included in the analysis if it followed similar inclusion criteria as in our study. The extraction was also performed independently by two authors (MJ and KS) and cross‐checked. The Newcastle‐Ottawa Scale (NOS) for assessing the quality of nonrandomized studies was used to assure the quality of included papers. Threshold of less than 12 stars was applied.

The study was approved by the Bioethics Committee of Medical University of Lodz.

## 3 RESULTS

Inclusion criteria were met by 389 patients; however, 67 were excluded from further analysis due to early loss in follow‐up. The final group of 322 patients is characterized in Table 1. In our study group, gender was not associated with smoking status (*P* = .92). Patients were significantly more often diagnosed because of hematuria than because of incidental finding during ultrasound examination (69% vs 31%; *P* < .01). In addition, gender was not associated with the size of the tumor (ie, a diameter of less than or more than 3 cm; *P* = .29) or with the existence of multiple tumors (*P* = 1.00). Similarly, smoking status was not associated with multiple tumors (*P* = .70) or size of the tumor (*P* = .39). Patients of different gender (*P* = .29) and of different smoking status (*P* = .93) presented with a similar T stage in TNM. Smokers and nonsmokers presented with similar tumor grading (*P* = .62) and were classified to similar risk groups (*P* = .86).

**Table 1:** Group description

Feature	Details			
Predictors				
Gender	Males: 74% (N = 237)		Females: 26% (N = 85)	
EAU risk group	Low: 37% (N = 119)	Medium: 26% (N = 83)		High: 37% (N = 120)
Age	Mean: 67.27 ± 11.14 years, Median: 68 years			
Smoking	Nonsmokers: 70% (N = 224)		Smokers: 30% (N = 98)	
T stage:	Ta: 63% (N = 203)	Tis: 3% (N = 9)		T1: 34% (N = 110)
Grading	G1: 54% (N = 174)	G2: 35% (N = 113)		G3: 11% (N = 35)
Number of tumors	Multiple: 36% (N = 115)		Single: 64% (N = 207)	
Diameter	Less than 3 cm: 70% (N = 226)		3 or more cm: 30% (N = 96)	
Outcomes				
BCG treated	Yes: 29% (N = 92)		No: 71% (N = 230)	
EORTC recurrence risk group	1‐year 15%/5‐year 31% risk: 31% (N = 100)	1‐year 24%/5‐year 26% risk: 37% (N = 119)	1‐year 38%/5‐year 62% risk: 32% (N = 102)	1‐year 61%/5‐year 78% risk: ~0% (N = 1)
EORTC progression risk group	1‐year 0.2%/5‐year 0.8% risk: 39% (N = 125)	1‐year 1%/5‐year 6% risk: 30% (N = 95)	1‐year 17%/5‐year 45% risk: 4% (N = 14)	1‐year 5%/5‐year 17% risk: 27% (N = 88)
CUETO recurrence risk group	1‐year 8.2%/5‐year 21% risk: 76% (N = 244)	1‐year 12%/5‐year 36% risk: 20% (N = 64)	1‐year 25%/5‐year 48% risk: 4% (N = 12)	1‐year 42%/5‐year 68% risk: 1% (N = 2)
CUETO progression risk group	1‐year 1.2%/5‐year 3.7% risk: 77% (N = 247)	1‐year 3%/5‐year 12% risk: 16% (N = 50)	1‐year 5.5%/5‐year 21% risk: 8% (N = 25)	1‐year 14%/5‐year 34% risk: 0% (N = 0)

Note

The table presents the general percentages of patients with partnonicular features, providing also the number of patients (N) with particular characteristics in our cohort.

**Figure 1:**
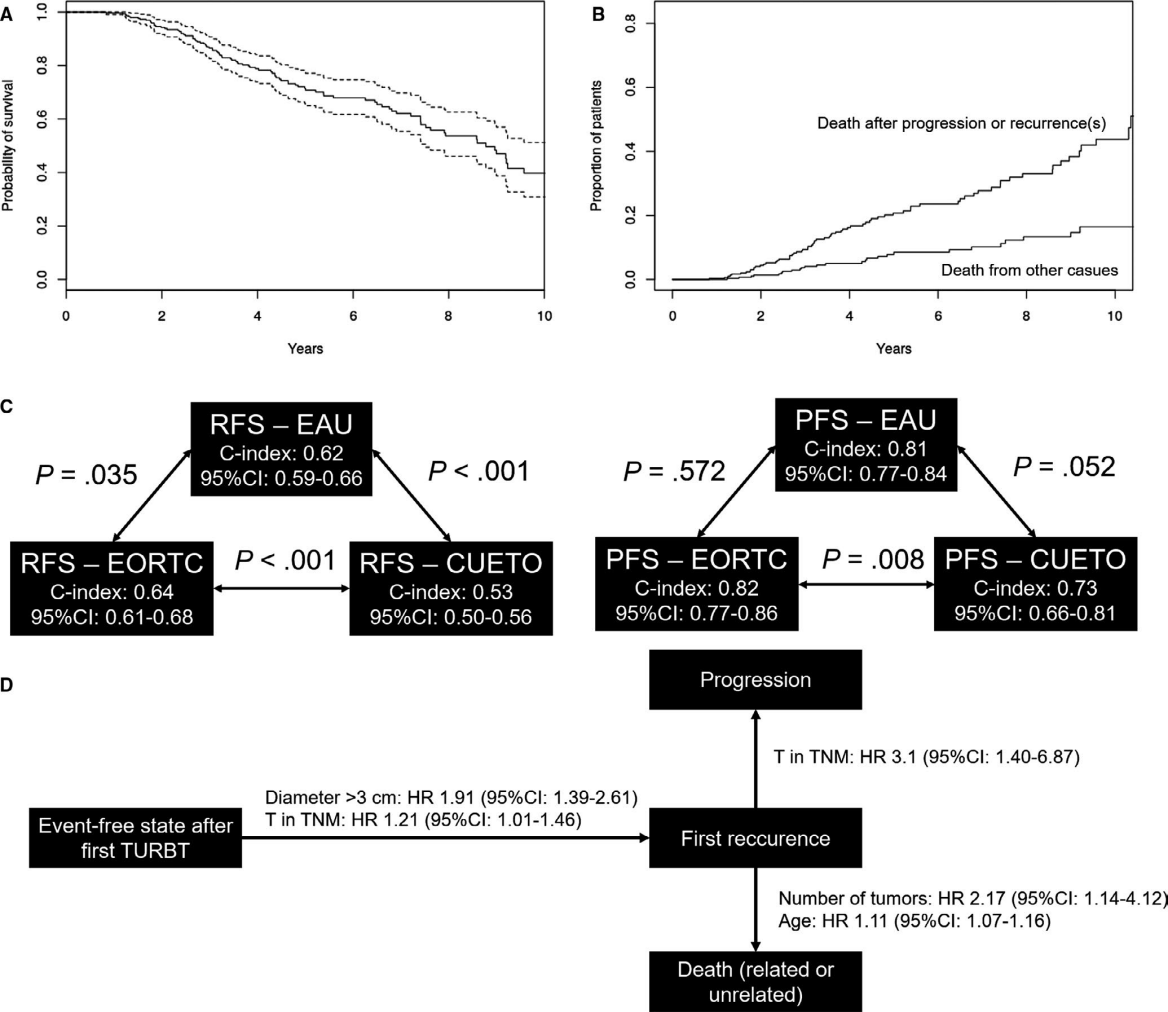
Overall survival of patients with diagnosed non–muscle‐invasive bladder cancer (NMIBC). Panel A represents the Kaplan‐Meier curve of overall survival in study group with its 95% confidence interval marked as dashed lines. Panel B presents the difference in proportion of patients dying after progression or recurrence and from other or uncertain causes in competing risk survival model. Panel C presents the overall c‐index values for recurrence and progression prediction using selected scoring models with their 95% confidence interval (95% CI). Presented *P*‐values represent the difference between c‐index calculated using one‐sample weighted student's *t* tests. Panel D represents the significant risk factors for transition from particular states in multistate Cox‐Markov survival model

Median follow‐up time was 48 months, with a maximum of 137 months. During that time 201 patients (62%; 95 CI: 57%‐68%) experienced at least one recurrence and 40 patients (12%; 95% CI: 9%‐17%) progressed. Neither of these results were greater than the lifetime risk reported in EAU guidelines (recurrence: vs ≥70%, *P* = .99; progression: vs ≥15%, *P* = .92). Median overall survival (OS) since the first diagnosis of NMIBC was 8.78 years (95% CI: 7.52‐10.31; Figure 1A) with the greater cumulative incidence of death after recurrence or progression (Figure 1B). Median recurrence‐free survival (RFS) was 2 years (95% CI: 1.25‐2.00) and median progression‐free survival (PFS) was not reached (due to competing risk of death from other cause).

As shown in Table 2, even though gender is used in the CUETO scoring system, it did not show any significant association with survival in univariate analysis. Existence of carcinoma in situ was not associated with modified RFS, PFS, or OS. A diameter of greater than 3 cm did shorten the RFS and PFS, but not OS. It was also noticeable that hematuria was significantly associated with shorter PFS and OS, despite it not being used in any of the studied systems.

**Table 2:** Risk factors of NMIBC recurrence, progression, and death in univariate Cox regression analysis

Factors present at the time of first TURBT procedure:	Univariate analysis									Used by		
	RFS			PFS			OS			EORTC	CUETO	EAU
	HR	95% CI	*P*	HR	95% CI	*P*	HR	95% CI	*P*			
Males vs females	1.12	0.82‐1.53	.47	1.45	067‐3.15	.35	1.33	0.81‐2.172	.26	No	Yes	No
Age at diagnosis (increase by 1)	1.01	0.99‐1.02	.42	1.04	1.01‐1.1	.01	1.08	1.05‐1.1	<.01	No	Yes	No
Nonsmokers vs tobacco smokers	0.81	0.6‐1.1	.17	0.95	0.48‐1.86	.88	0.66	0.41‐1.05	.08	No	No	No
Tis vs Ta	1.38	0.61‐3.13	.45	8.76	0.91‐84.35	.06	2.63	0.63‐10.92	.18	Yes	Yes	Yes
T1 vs Ta	2.11	1.59‐2.8	<.01	26.92	8.29‐87.48	<.01	2.13	1.43‐3.18	<.01	Yes	Yes	Yes
G2 vs G1	1.47	1.09‐1.99	<.01	8.74	3.32‐23.03	<.01	2.34	1.52‐3.6	<.01	Yes	Yes	Yes
G3 vs G1	2.54	1.69‐3.84	<.01	15.45	5.3‐45.03	<.01	2.75	1.5‐5.05	<.01	Yes	Yes	Yes
Multiple vs single tumor	1.7	1.28‐2.24	<.01	2.63	1.4‐4.92	<.01	1.69	1.13‐2.52	.01	Yes	Yes	Yes
Diameter >3cm vs <3 cm	2.15	1.61‐2.85	<.01	3.12	1.68‐5.83	<.01	1.25	0.82‐1.92	.3	Yes	No	Yes
Hematuria vs no hematuria	1.09	0.81‐1.47	.55	2.30	1.02‐5.19	<.05	1.96	1.22‐3.14	<.01	No	No	No

Note

Underlined feature is considered as a reference in HR computation.

Abbreviations: 95% CI, 95% confidence interval; HR, hazard ratio; OS, overall survival; PFS, progression‐free survival; RFS, recurrence‐free survival.

Due to the long duration of accrual and follow‐up, changes in guidelines, and high rate of consent withdrawal (48%), the BCG therapy was administered mostly, but not only, to patients in the high‐risk modern EAU group (*P* < .01; 58 patients, 48%). Exactly 16 patients (19%) of medium‐ and 18 patients (15%) of low‐risk group were also treated with BCG.

Among the patients in high‐risk subgroup (N = 120), those who did not withdraw their consent and were treated with BCG tended to be younger (mean 66.4 years vs 73.3 years; *P* < .01) and smokers (23/35 vs 12/50; *P* = .02). While BCG therapy did not influence the RFS (HR 1.09; 95% CI: 0.81‐1.46) or PFS in the group as a whole (HR 0.55; 95% CI: 0.25‐1.21), this effect was noticeable in the high‐risk group (for RFS: HR 0.50; 95% CI: 0.33‐0.76; for PFS: HR 0.16; 95% CI: 0.07‐0.40). No such observation was noted for the OS, where BCG therapy extended the life of high‐risk patients (HR 0.18; 95% CI: 0.09‐0.37) and those from all risk groups (HR 0.47; 95% CI: 0.29‐0.74).

### 3.1 EAU, EORTC, and CUETO risk groups

**Figure 2:**
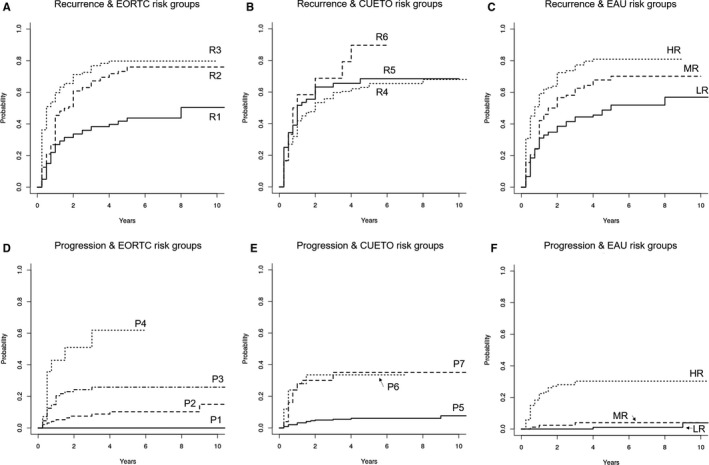
Cumulate incidence plots of recurrence and progression among patients in specific risk strata. Percent values are given to describe the plots in panels A‐D are given for expected incidence of 1‐ and 5‐year recurrence or progression rates. Panel E and F represent the utility of EAU risk groups as described in guidelines. Cumulative incidence of death (competing risk) in subgroups as well as risk groups of 3 or fewer patients were discarded to enhance readability. R1—EORTC 1‐year 15% and 5‐year 31% risk of recurrence, R2—EORTC 1‐year 24% and 5‐year 46% risk of recurrence, R3—EORTC 1‐year 38% and 5‐year 62% risk of recurrence, R4—CUETO 1‐year 8.2% and 5‐year 21% risk of recurrence, R5—CUETO 1‐year 12% and 5‐year 36% risk of recurrence, R6—CUETO 1‐year 25% and 5‐year 48% risk of recurrence, P1—EORTC 1‐year 0.2% and 5‐year 0.8% risk of recurrence, P2—EORTC 1‐year 1% and 5‐year 6% risk of recurrence, P3—EORTC 1‐year 5% and 5‐year 17% risk of recurrence, P4—EORTC 1‐year 17% and 5‐year 45% risk of recurrence, P5—CUETO 1‐year 1.2% and 5‐year 3.7% risk of recurrence, P6—CUETO 1‐year 3% and 5‐year 12% risk of recurrence, P7—CUETO 1‐year 5.5% and 5‐year 21% risk of recurrence, LR—EAU low‐risk group, MR—EAU medium‐risk group, and HR—EAU high‐risk group

As shown in Figure 1C, the EORTC model was superior to EAU and CUETO model in predicting recurrence. For progression prediction, EORTC performed better than CUETO but there was no sufficient evidence that it also performed better than EAU. Noteworthy, all c‐indices for progression prediction were greater than for recurrence. The cumulative incidences in different risk groups are shown in Figure 2.

Risk stratification of overall survival using EAU‐based groups lead to overall concordance (c‐index) of 0.64; with 0.82 for 1‐year and 0.65 for 5‐year prediction. A similar observation was made for RFS (1‐year c‐index: 0.64; 5‐year: 0.62), where medium‐ and high‐risk groups were also associated with shorter RFS in comparison with low‐risk group, as shown in Table 2. EAU risk groups used as predictors had high model concordance for progression and death (Table 3).

**Table 3:** Concordance of EAU risk group stratification

	Recurrence		Progression		Survival	
	Overall	With BCG	Overall	With BCG	Overall	With BCG
1‐year c‐index	0.639	0.560	0.811	0.696	0.815	—
5‐year c‐index	0.631	0.540	0.785	0.635	0.651	0.608

Note

The table provides Harrell's c‐index for right‐censored event times derived from Cox regression models developed for particular analysis.

As expected, each additional EORTC scoring point (HR: 1.19; 95% CI: 1.14‐1.26) and CUETO point (HR: 1.07; 95% CI: 1.01‐1.14) were associated with shortened RFS. The same was observed for progression (PFS, EORTC HR: 1.234; 95% CI: 1.16‐1.31; CUETO HR: 1.64; 95% CI: 1.41‐1.91). After conversion to the defined four risk groups, we have calculated the c‐index values comparing the concordance of reference risk stratification to our group. Results of this have been presented in Table 3.

To assess further the predictive abilities of reference scores and risk groups, we performed receiver operating characteristic (ROC) curve analysis for recurrence and progression in 1‐ and 5‐year periods (Figure S1). The areas under the ROC curve (AUC) ranged from 0.46 to 0.69 for prediction or recurrence and from 0.66 to 0.82 for progression (Table 5).

Our group consisted of 58 EAU high‐risk patients that we treated with BCG. In this group, EORTC achieved a concordance of 0.56 (95% CI: 0.48‐0.63) for recurrence prediction, whereas CUETO achieved a concordance of 0.57 (95% CI: 0.50‐0.65) and was not significantly different from EORTC (*P* = .69). For progression, the EORTC system yielded a c‐index of 0.66 (95% CI: 0.35‐0.98) and CUETO showed 0.55 (95% CI: 0.23‐0.86). The difference between those two models was not statistically significant (*P* = .48), meaning that both EORTC and CUETO showed low and surprisingly comparable performance in EAU high‐risk patients who were treated with BCG.

### 3.2 Factors affecting transition

The developed Cox‐Markov model highlighted the importance of tumor diameter and extent of primary tumor in the development of first progression (Figure 1D). Extent of primary tumor was also shown to be an important factor for shortening the time from first recurrence to progression (Table S3).

### 3.3 Systematic review

To further confirm our observations, a systematic review was performed. Designed search query identified 176 publications for screening. However, 40 publications were discarded as reviews, 77 due to another study questions and 24 due to another study groups. This resulted in 35 papers being included in full‐text analysis, following which another 17 were excluded because of lack of appropriate analysis (no analysis of concordance) and two due to following other study questions. None of the studies dropped out in quality analysis. Detailed results of this process were included in Table S1. C‐indices extracted from the final 16 publications were appended to Table 4 and AUC values to Table 5. Mean values presented in the tables indicate that all of these methods generally perform better for progression prediction, and the lower concordance in patients treated with BCG in rather universal, even in CUETO system (where the c‐index improvement was often marginal).

**Table 4:** Concordance indices (c‐index) for application of EORTC and CUETO risk stratification models with results of systematic review

		EORTC				CUETO			
		Recurrence		Progression		Recurrence		Progression	
		Overall	With BCG	Overall	With BCG	Overall	With BCG	Overall	With BCG
**This study**	**1‐year c‐index**	**0.657**	**0.589**	**0.820**	**0.718**	**0.557**	**0.580**	**0.760**	**0.738**
	**5‐year c‐index**	**0.649**	**0.570**	**0.809**	**0.681**	**0.547**	**0.559**	**0.715**	**0.574**
Fernandez‐Gomez et al (2011)^36^	Overall c‐index	0.630	—	—	—	—	—	—	—
Sylvester et al (2006)^5^	1‐year c‐index	0.660	—	0.740	—	—	—	—	—
	5‐year c‐index	0.660	—	0.750	—	—	—	—	—
Fernandez‐Gomez et al (2009)^6^	1‐year c‐index	—	—	—	—	0.636	—	0.687	—
	5‐year c‐index	—	—	—	—	0.644	—	0.700	—
Xylinas et al (2013)^17^	Overall c‐index	0.597	0.554	0.662	0.576	0.523	0.597	0.616	0.645
Ravvaz et al (2017)^37^	1‐year c‐index	0.630	0.570	0.790	0.710	0.590	0.560	0.790	0.640
	5‐year c‐index	0.590	0.530	0.740	0.690	0.560	0.570	0.720	0.610
Tianyuan et al (2013)^38^	Overall c‐index	0.711	—	0.768	—	0.663	—	0.741	—
Dalkilic et al (2018)^39^	5‐year c‐index	0.777	0.823	0.801	0.832	0.705	0.758	0.881	0.806
Pillai et al (2011)^40^	1‐year c‐index	0.620	—	0.650	—	—	—	—	—
	5‐year c‐index	0.630	—	0.670	—	—	—	—	—
Almeida et al (2015)^41^	1‐year c‐index	0.700	—	—	—	—	—	—	—
	5‐year c‐index	0.720	—	—	—	—	—	—	—
Busato Junior et al (2015)^42^	1‐year c‐index	—	—	0.860	—	—	—	—	—
	5‐year c‐index	—	—	0.780	—	—	—	—	—
Choi et al (2014)^43^	Overall c‐index	0.759	—	0.704	—	0.836	—	0.745	—
Vedder et al (2014)^44^ *Spain*	Overall c‐index	0.590	—	—	—	0.590	—	—	—
Vedder et al (2014) *Denmark*	Overall c‐index	0.610	—	—	—	0.560	—	—	—
Vedder et al (2014) *The Netherlands*	Overall c‐index	0.550	—	—	—	0.580	—	—	—
Mean c‐index (based on all reported c‐indices)		0.652 ± 0.059	0.606 ± 0.099	0.753 ± 0.061	0.701 ± 0.075	0.615 ± 0.081	0.604 ± 0.070	0.736 ± 0.066	0.669 ± 0.079

Note

C‐index is given as a fraction with 1.0 indicating maximum concordance. The parameters not provided in the analyzed manuscripts were marked with em‐dashes.

**Table 5:** Area under the receiver operating characteristic (ROC) curves shows moderate diagnostic utility of selected models

		EAU risk groups	CUETO score	CUETO risk groups	EORTC score	EORTC risk groups
*Recurrence*						
This study	1‐year RFS	0.633	0.566	0.461	0.670	0.646
	5‐year RFS	0.652	0.539	0.484	0.693	0.678
Kılınç et al (2017)^45^—5‐year RFS			—	—	—	0.773
Hernandez et al (2011)^46^—1‐year RFS			—	—	—	0.61
Hernandez et al (2011)—5‐year RFS			—	—	—	0.70
Choi et al (2014)^43^—5‐year RFS			0.894	—	0.832	—
*Progression*						
This study	1‐year PFS	0.821	0.674	0.770	0.803	0.801
	5‐year PFS	0.805	0.664	0.728	0.802	0.788
Kılınç et al (2017)—5‐year PFS			—	—	—	0.901
Hernandez et al (2011)—1‐year PFS			—	—	—	0.58
Hernandez et al (2011)—5‐year PFS			—	—	—	0.55
Choi et al (2014)—5‐year PFS			0.724	—	0.722	—

Note

The sensitivity and specificity metrics were calculated for each point as a threshold. ROC curves for our subgroup were shown in Figure S1.

## 4 DISCUSSION

This study validates the use of EORTC, CUETO, and EAU risk stratification algorithms in the prediction of recurrence, progression, and death of patients with newly diagnosed NMIBC. Our analysis included 322 patients and confirmed observations from previous studies in terms of intragroup associations,^10^, ^11^ indicating that our study group is representative.

The EORTC model demonstrated superior performance, although this performance is generally moderate. The developed multistate model depicted the role of the extent, diameter, and number of tumors in their recurrence, progression, and, finally, death. The systematic review confirmed that tools for risk stratification are insufficient for validation in a real clinical scenario. The study includes a comprehensive assessment of not only the simplified risk groups presented in EAU, EORTC, and CUETO publications but also the score that is used for the development of these risk groups.

Risk stratification algorithms for NMIBC are in great demand as the progression to MIBC is associated with poor prognosis; this was confirmed not only by our analysis but also by several others.^12^ Despite the known risk factors and continuous repetition of TURBT procedures, the accuracy of recurrence and progression to MIBC is still unsatisfactory. Our results and systematic review confirm that state‐of‐the‐art risk stratification tools demonstrate poor discriminative abilities in forecasting both recurrence and progression; however, the latter seems to be more accurately predicted.

Our findings are first to confirm that EORTC offers a significant advantage over EAU and CUETO in recurrence prediction. No previous study has compared c‐indices directly with their 95% CI. However, even though this superiority may be not relevant from the clinical point of view, because the c‐index values are generally low, it may still be relevant to the progression prediction. Although EAU and EORTC did not display any significant difference in this regard, EORTC was found to be superior to CUETO. It is important to remember that CUETO was initially developed for BCG‐treated patients.

Although the discussed systems could be used to assess prognosis in recurrent cases, our study only analyzed survival to the first recurrence. The individual surgeon has a significant impact on the risk of recurrence after curative treatment of patients with NMIBC, as described previously,^13^ and another approach could aggravate the lead‐time bias. A similar reasoning has been adopted in several similar studies ^14^

Our systematic analysis also demonstrates the inconsistency in reporting the validity of utilized stratification approaches. For example, neither the very recent analysis of 301 patients by Wang et al^15^ nor an analysis of 1436 patients, a group without immediate postoperative instillation of chemotherapy, by Rieken et al^16^ could be included in the review due to lack of c‐index or AUCROC analysis. Even if the c‐index values are reported, they are usually provided without 95% confidence interval, hence are unfit for meta‐analysis. Nevertheless, most of the cited papers indirectly confirm our observations: the accuracy of predictions was consistently decreased in patients treated with BCG in all included publications.^17^

Together with the completion of our analysis at the end of 2018, a critical assessment from the European Association of Urology Non–muscle‐invasive Bladder Cancer Guidelines Panel was published.^11^ The paper concluded that none of the available risk stratification and prognostic models reflects current standards of treatment. It proposes that the EORTC risk tables and CUETO scoring model should be updated with previously unavailable data and recalculated. Our data support this conclusion.

Multiple discrepancies between original publications and validation studies are reported in the reviewed material. For example, patients requiring second TURBT were dropped from the analysis in original CUETO and EORTC publications, while multiple recent papers did not secure this criteria.^7^

Despite numerous attempts to develop new models, it was only recently that c‐indices were determined for the 5‐year recurrence (0.65) and progression (0.70).^18^ Considering the risk of overfitting of the model (c‐index provided without external validation) and the fact that our validation found EORTC to provide similar c‐indices, its utility requires further extensive validation. Similarly, Hong et al^19^ report an AUCROC of proposed nomogram as 0.604 for the 5‐year prediction of recurrence. In our study, without utilization of proposed nomograms, better validation AUCROC metrics were achieved by EAU risk groups, EORTC score, and risk groups. Moreover, a recently proposed model for patients treated with 1‐3 years of maintenance BCG,^20^ based only on grading and age, was described; this demonstrated a c‐index of 0.59 for training and 0.56 for validation sets for recurrence. Those values were covered by 95% CI for c‐indices provided in this study; these were given for mixed population of both patients treated with BCG and not. A similar situation was noted for progression, where the authors provided c‐indices of 0.72 and 0.64 for training and validation sets, respectively.

NMIBC treatment is still evolving: different stages and grades need appropriate treatment and follow‐up procedures. Current guidelines regarding operative and intravesical treatment remain inconsistent in some areas.^21^ TURBT is still an irreplaceable tool for removing and evaluating tissue resected from the bladder, despite different operation times and sequences. While BCG immunotherapy remains the most widely chosen, safe, and appropriate regimen for intravesical treatment, Mitomycin C (MMC) chemotherapy, which is instilled to the bladder within 24 hours post‐TURBT, is also commonly used.^22^ In addition, while a single instillation of epirubicin, gemcitabine, or pirarubicin have also shown valuable effects, no randomized comparisons of individual drugs have been conducted.^22^, ^23^, ^24^ Randomized control trials have yielded unsatisfying results for BCG plus MMC,^25^ interferon plus BCG,^26^ and interferon plus epirubicin combination therapy.^27^

As so few new regimens exist for intravescal treatment, current trends for development are currently targeting delivery systems. The three best‐known devices are based on hyperthermia to the bladder wall, circulating chemotherapy, and ionization of chemotherapy. Electromotive Drug Administration EMDA^®^‐MMC enhances the delivery of chemotherapy by electro‐osmosis, ionophoresis, and electroporation.^28^ A randomized controlled trial has found MMC to be an effective way of implementing EMDA and BCG in patients with high‐risk tumors.^29^ Although many studies have been performed with EMDA, the level of evidence was low and the time to recurrence and progression or side effects remain incomplete, suggesting more studies are needed.^30^

Many trials have been performed using monoclonal antibodies in the treatment of urothelial carcinoma.^31^, ^32^ One such IgG1 monoclonal antibody is durvalumab; which has been found to binding with high affinity to the PD‐L1 receptor. In NMIBC, durvalumab is added to BCG immunotherapy intravenously. Although durvalumab is currently under evaluation for the treatment of BC in a number of trials, only preliminary reports have so far indicated its role in BC treatment.^31^, ^33^, ^34^

Lastly, it is worth mentioning that current risk stratification tools are hard to apply in the field of personalized medicine. For example, based on a standard cutoff of 50%, classification of 38% probability for 1‐year recurrence, and 62% probability for 5‐year recurrence into EORTC would yield 38% incorrect predictions for both timeframes. Currently available nomograms do not predict expected time of recurrence or progression, and hence, cannot be treated as predictive tests for particular patients. This means that despite description of general predictive potential using AUCROC or c‐index parameters, analysis of these tools as predictive models in terms of their accuracy, sensitivity, or specificity is futile.

Our study is not devoid of limitations associated with study design. As a retrospective analysis, possible recall and selection bias should be considered. To counter this, the results were integrated with data received from a central registry; however, only overall survival was analyzed using the information from the central governmental registry. The data about recurrence and progression were obtained only from one facility, which was not the only provider of TURBT procedures in the region: some patients may have chosen different facilities for further treatment and these were lost in follow‐up, and the procedures were performed by multiple surgeons and were assessed by multiple pathologists. Comorbidities might also have an uncontrolled influence on treatment and decision making, however, based on our findings, our sample appears representative for the population. Additionally, none of the patients was treated with immediate single intravesical instillation of gemcitabine. However, the recent evidence suggests that this further decreases the predictive performance of studied systems.^35^

Nevertheless, our study provides additional evidence regarding the validity of risk stratification based on EAU guidelines on a fairly large sample. It is also the first to summarize current research and compare all three currently recommended methods of risk assessment. Individual patient data have been anonymized and shared to facilitate further research in the area.

In conclusion, EAU, EORTC, and CUETO risk groups appear to demonstrate moderate performance in the prediction of recurrence and progression. Combined with recent advancements in treatment options, those results jointly highlight the urgent need for the development of new stratification tools. For patients treated with BCG, and without, and without any immediate postoperative chemotherapy, EORTC was shown to perform better in predicting recurrence and progression than CUETO; however, EORTC demonstrated no superiority over EAU.

## CONFLICT OF INTEREST

None declared.

## AUTHORS' CONTRIBUTION

Jobczyk—protocol/project development, data collection or management; Stawiski—protocol/project development, data collection or management, data analysis, manuscript writing/editing; Fendler—protocol/project development, manuscript writing/editing; Różański—protocol/project development.

## COMPLIANCE WITH ETHICAL STANDARDS

Written informed consent was obtained from all patients before treatment, which allowed for anonymous retrospective analysis of collected data in concordance with Polish law. The study was approved by the local ethical committee at the Medical University of Lodz.

## Supporting information

Fig S1

Table S1

Table S2

Table S3

## Data Availability

Individual patient data are attached to this manuscript as Table S2.
