# Supplementary figures and images for: Validation of EORTC, CUETO, and EAU risk stratification in prediction of recurrence, progression, and death of patients with initially non–muscle‐invasive bladder cancer (NMIBC): A cohort analysis
Source: Cancer Med. 2020 Mar 26;9(11):4014–25. doi: 10.1002/cam4.3007 (PMC7286464; doi:10.1002/cam4.3007)

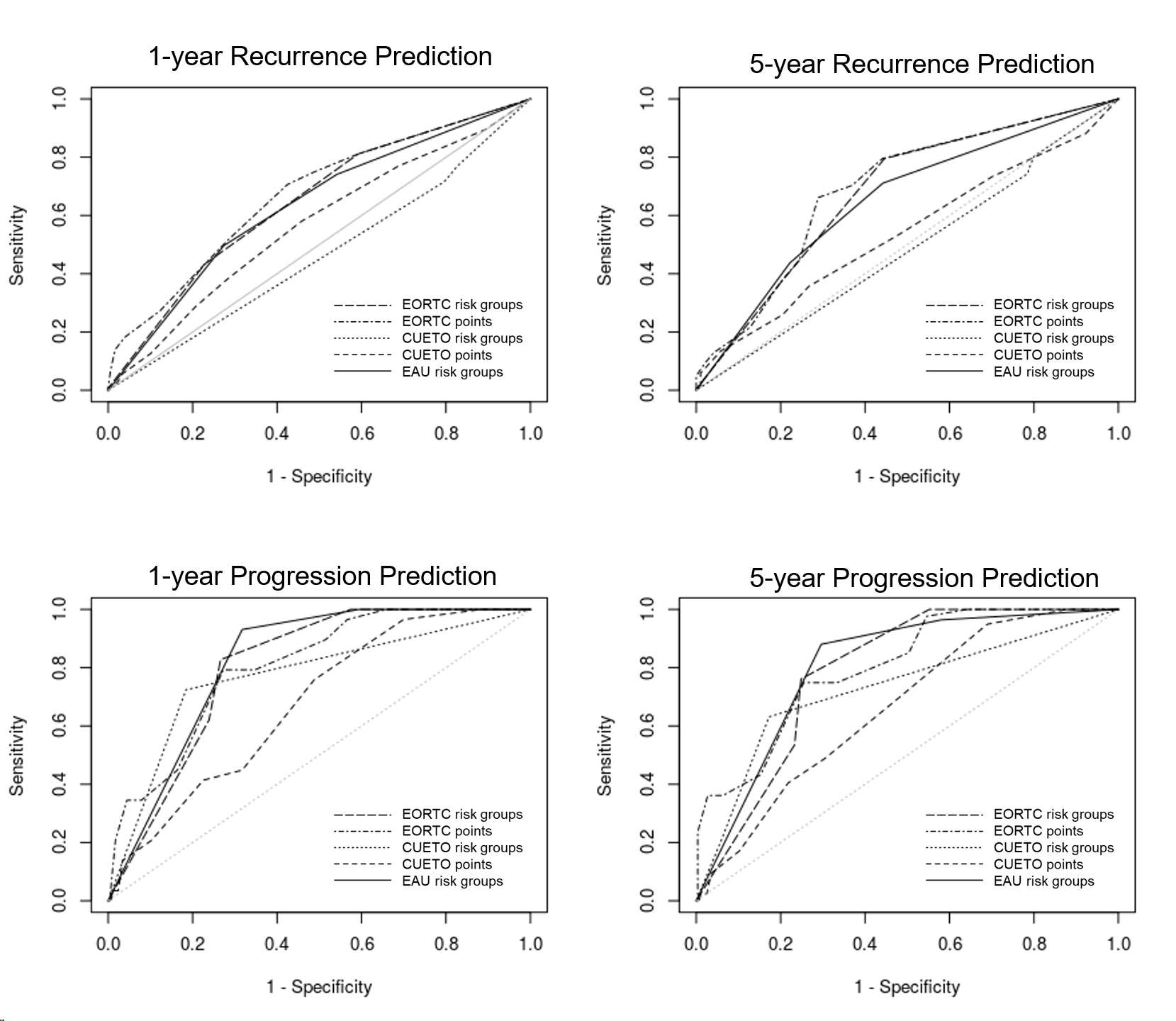

Supplement: Supplementary file 1 — Fig S1 [file CAM4-9-4014-s001.tif]
